# The Clinical Relevance of Pain Severity Changes: Is There Any Difference Between Asian and Caucasian Patients With Osteoarthritis Pain?

**DOI:** 10.1111/papr.12835

**Published:** 2019-11-20

**Authors:** Li Yue, Jianing Wang, Hiroyuki Enomoto, Shinji Fujikoshi, Levent Alev, Yan Yolanda Cheng, Vladimir Skljarevski

**Affiliations:** ^1^ Medical Department Lilly Suzhou Pharmaceutical Co. Ltd. Shanghai Branch Shanghai China; ^2^ Medicine Development Unit‐Japan Eli Lilly Japan K.K. Tokyo Japan; ^3^ Medicine Development Unit‐Japan Eli Lilly Japan K.K. Kobe Japan; ^4^ TR Medical Mgmt Eli Lilly Turkey Istanbul Turkey; ^5^ Lilly Research Laboratories Eli Lilly and Company Indianapolis Indiana U.S.A

**Keywords:** Asian patient, Caucasian patient, chronic pain, clinical improvement, duloxetine, osteoarthritis, pain severity, racial difference

## Abstract

The objective of the present analysis was to determine whether changes in Brief Pain Inventory (BPI) average pain scores by patient global impression of improvement (PGI‐I) category and the cut‐off for clinically important difference (CID) were different between Asian and Caucasian patients with chronic pain due to osteoarthritis. This analysis used data from 3 (Caucasian) and 2 (Asian) randomized, placebo‐controlled, 10‐ to 14‐week duloxetine studies for the treatment of patients ≥40 years of age with osteoarthritis pain. The receiver operating characteristic (ROC) analysis was used to characterize the association between changes in BPI average pain scores and PGI‐I levels at study endpoint. The CID was characterized by PGI‐I, and the cut‐off point for CID in BPI average pain scores was determined by the intersection of a 45‐degree tangent line with each ROC curve. Data from 668 Asian and 868 Caucasian patients were available for analysis. Baseline BPI average pain ratings including worst and least pain were comparable between Asians and Caucasians. Ratings for percentage change from baseline to endpoint for BPI average pain scores in Asian patients and Caucasian patients were similar across the 7 PGI‐I categories, regardless of age, gender, study, and treatment. The ROC analysis results of cut‐off points in BPI average pain scores demonstrated the raw change cut‐off was −3.0, and percentage change cut‐off was −40% for both Asian and Caucasian patients. Overall, the present analysis concludes changes in BPI average pain scores by PGI‐I category and the cut‐off for CID were similar for Asian and Caucasian patients with chronic pain due to osteoarthritis.

## Introduction

Osteoarthritis (OA) is one of the most prevalent chronic musculoskeletal disorders worldwide, especially in the elderly.^1^ It is a leading cause of deteriorated health‐related quality of life (HRQoL) due to chronic pain.^2^, ^3^, ^4^, ^5^ According to the Global Burden of Disease Study, the disease‐adjusted life years caused by OA increased by 105% from 1990 to 2016.^6^ Pain is recognized as one of the hallmark symptoms in OA and is the primary reason patients seek medical attention,^7^, ^8^, ^9^, ^10^ since pain severity has a greater impact on HRQoL than other joint symptoms in patients with OA.^8^

Ogawa et al.^11^ reported that improvement in pain was positively associated with improvement in quality of life. In most pain clinical trials, reduction in pain severity has been used as the primary outcome measure. Patients interpret measurement scales very differently when reporting pain, and baseline ratings can vary widely within a study population.^12^, ^13^ The Initiative on Methods, Measurement, and Pain Assessment in Clinical Trials (IMMPACT) recommends the use of the Patient Global Impression of Improvement (PGI‐I) scale as an outcome measure for chronic pain in clinical trials.^14^ The PGI‐I scale is used as an anchor to determine the importance of the pain rating to the patient in a manner that is self‐evident and readily interpretable.^14^

Clinical pain studies have demonstrated cultural variations in response to painful stimuli.^15^, ^16^ These differences in pain expression are important in clinical practice because they influence diagnosis and treatment.^17^ Factors such as ethnicity, gender, and personal opinions have marked influences on patient ratings of pain intensity.^13^, ^18^ Hobara examined the effects of the participants’ gender and culture on their expressions of pain.^18^ The study's results suggested that Asian participants (Japanese) place emphasis on stoicism and the desirability of concealing pain and emotions, whereas Caucasian participants (Euro‐American) place greater emphasis on the expression of personal feelings.^18^, ^19^

Duloxetine is a potent and selective inhibitor of serotonin and norepinephrine in the central nervous system and is efficacious for chronic musculoskeletal pain.^20^, ^21^, ^22^ The pain inhibitory action of duloxetine is believed to be due to its effect in enhancing descending pain inhibitory systems.^20^, ^21^, ^22^ Duloxetine has demonstrated clinically meaningful reductions in average pain ratings relative to placebo among patients with chronic OA knee pain in 3 double‐blind, randomized, placebo‐controlled clinical trials.^20^, ^21^, ^22^ A clinically important difference (CID) in Brief Pain Inventory (BPI) average pain severity scores, using the PGI‐I as an anchor, has been previously established in 5 randomized, placebo‐controlled, duloxetine studies and found to be consistent across age groups, gender, and pain types.^21^ Also, CID cut‐off points for least, average, and worst pain are well established in duloxetine studies.^21^ However, there were no analyses examining potential racial differences between Asian and Caucasian patients with chronic pain. Hence, the objective of the present analysis was to determine if the changes in BPI average pain scores by PGI‐I category and the cut‐off for CID were different for Asian patients when compared to Caucasian patients with OA of the knee or hip in duloxetine studies.

## Methods

This analysis used data from 3 (Caucasian)^21^, ^22^, ^23^ and 2 (Asian)^24^, ^25^ randomized, placebo‐controlled, parallel, multicenter, 10‐ to 14‐week, OA pain studies of duloxetine (20, 40, 60, and 120 mg once daily). Both men and women ≥40 years of age with pain arising from knee or hip OA were included. Patients enrolled in these studies were required to have a pain severity score of at least 4 (moderate pain severity) on the BPI 24‐hour average pain item. The BPI is a validated self‐reported questionnaire that assesses pain severity using the numeric rating scale for pain intensity (NRS‐PI, 0 to 10 scale, where 0 = no pain, 4 to 6 = moderate pain, and 10 = worst possible pain) for the conditions of worst, least, and average pain, as well as “pain right now.”^13^ The investigational review board of each clinical trial site approved the study protocol, and studies were conducted in accordance with the principles of the Declaration of Helsinki. Written informed consent was obtained from each patient prior to participation in any study‐related procedures. All 5 studies used similar study designs, procedures, outcome measures, and assessment tools, including well‐validated BPI^26^, ^27^ ratings to assess the patient's worst, least, and average pain intensity over the preceding 24 hours. These studies also used the PGI‐I scale to assess the patient's perceived improvement of pain. The PGI‐I is a 1‐item, 7‐point scale that allows the patient to characterize his or her own opinion about overall change from the time of randomization to the end of the trial. The scale ranges from 1 (very much better) to the midpoint of 4 (no change) to 7 (very much worse).^27^ The BPI and PGI‐I are the commonly used assessment tools in pain clinical trials, and both are recommended by IMMPACT to interpret the clinical importance of treatment outcomes in chronic pain clinical trials.^14^ Both BPI and PGI‐I assessment tools are self‐administered questionnaires, thus reducing the possibility of any caregiver or investigator influence on the results. The PGI‐I has been effectively used as a secondary outcome measure in several pain clinical studies, which demonstrates its reliability and validity as an outcome measure.^28^, ^29^, ^30^, ^31^

In all studies, the BPI ratings for worst, least, and average pain intensity were collected at baseline, and the BPI average pain and the PGI‐I ratings were collected at several post‐baseline visits. The analysis was carried out using the values collected at the last visit of the double‐blind treatment phase. The percentage change from baseline in BPI average pain severity using the 0‐to‐10 numeric scale compared to the 7‐category PGI‐I assessment at study endpoint was computed for each patient. The relationship between percentage change in BPI scores and the endpoint PGI‐I scores was plotted by displaying the BPI average change within each PGI‐I category. Two categories on the PGI‐I scale, “very much worse” or “much worse,” were combined because of the small numbers of patients in these groups. Reduced calculations for BPI average pain scores from baseline to endpoint by PGI‐I category, according to race (Asian and Caucasian) and study participation, were evaluated. In addition, subgroup analyses by age groups (≥40 to <50; ≥50 to <60, ≥60 to <70, and ≥ 70 years), gender (males vs. females), and study treatment group (duloxetine vs. placebo) were performed to explore whether they influence or confound PGI‐I distribution between the races. Moreover, the percentage change from baseline in BPI average pain scores by PGI‐I category according to race (Asian and Caucasian) in duloxetine‐treated patients was assessed. All comparisons were made using descriptive statistics. The percentage change data are summarized in graphical format, while results from receiver operating characteristic (ROC) analysis are presented in tabular format.

In the present analysis, 3 different levels of improvement as characterized by the PGI‐I were examined: “a little better” or higher (PGI‐I score = 1, 2, or 3); “much better” or higher (PGI‐I score = 1 or 2); and “very much better” (PGI‐I score = 1). CID was characterized by the PGI‐I and was defined as “much better” or “very much better.” The ROC curves were generated by computing the sensitivity and specificity in a contingency table (2 × 2) for both the raw and percentage changes for BPI average pain scores, and for each level of the PGI‐I, using the proportion of patients who reported each possible level of change in pain rating as the exposure and the preselected level of the PGI‐I as the outcome. The appropriate cut‐off point for the BPI average pain percentage change was determined using ROC analysis. ROC analysis is a standard statistical analysis used to best characterize the association between change in the BPI average pain score (independent variable) and different levels of the PGI‐I (dependent variable) at the study endpoint. The cut‐off point for CID was determined by the intersection of a 45‐degree tangent line with each ROC curve; thus, the pain intensity changes can be best associated with each of the categories of improvement on the PGI‐I.

## Results

Data from 668 Asian and 868 Caucasian patients, who had recorded baseline pain intensity and at least 1 post‐baseline measure on the BPI and PGI‐I, were included in the analyses. A summary of patient demographics and baseline characteristics of the combined studies is presented in Table 1. In the present analysis, baseline BPI average pain ratings based on the raw ratings were similar between Asian and Caucasian patients. In addition, baseline BPI ratings for worst and least pain were comparable between Asian and Caucasian patients.

**Table 1:** Patient Demographics and Baseline Characteristics

	Asian Studies		Caucasian Studies		
	Study 1 (*N *=* *407)	Study 2 (*N* = 261)	Study 3 (*N* = 194)	Study 4 (*N* = 250)	Study 5 (*N* = 424)
Gender, *n* (%)					
Female	311 (76.4)	207 (79.3)	126 (64.9)	191 (76.4)	237 (55.9)
Male	96 (23.6)	54 (20.7)	68 (35.1)	59 (23.6)	187 (44.1)
Age, median years (25th to 75th percentile)	61.1 (55.0 to 66.3)	66.0 (61.0 to 72.0)	62.7 (55.0 to 70.3)	62.5 (55.8 to 70.0)	60.8 (55.1 to 67.3)
Race, *n* (%)					
Asian	407 (100)	261 (100)	0	0	0
Caucasian	0	0	194 (100)	250 (100)	424 (100)
BPI worst pain rating, median (25th to 75th percentile)	7.0 (6.0 to 8.0)	7.0 (6.0 to 7.0)	8.0 (7.0 to 8.0)	8.0 (7.0 to 8.0)	8.0 (6.0 to 8.0)
BPI least pain rating, median (25th to 75th percentile)	4.0 (2.0 to 5.0)	3.0 (2.0 to 4.0)	5.0 (3.0 to 6.0)	5.0 (3.0 to 6.0)	4.0 (3.0 to 6.0)
BPI average pain rating, median (25th to 75th percentile)	5 (4.0 to 6.0)	5.0 (4.0 to 6.0)	6.0 (5.0 to 7.0)	6.0 (5.0 to 7.0)	6.0 (5.0 to 7.0)

BPI, Brief Pain Inventory; *N*, number of subjects in analysis population; *n*, number of subjects in each category.

**Figure 1:**
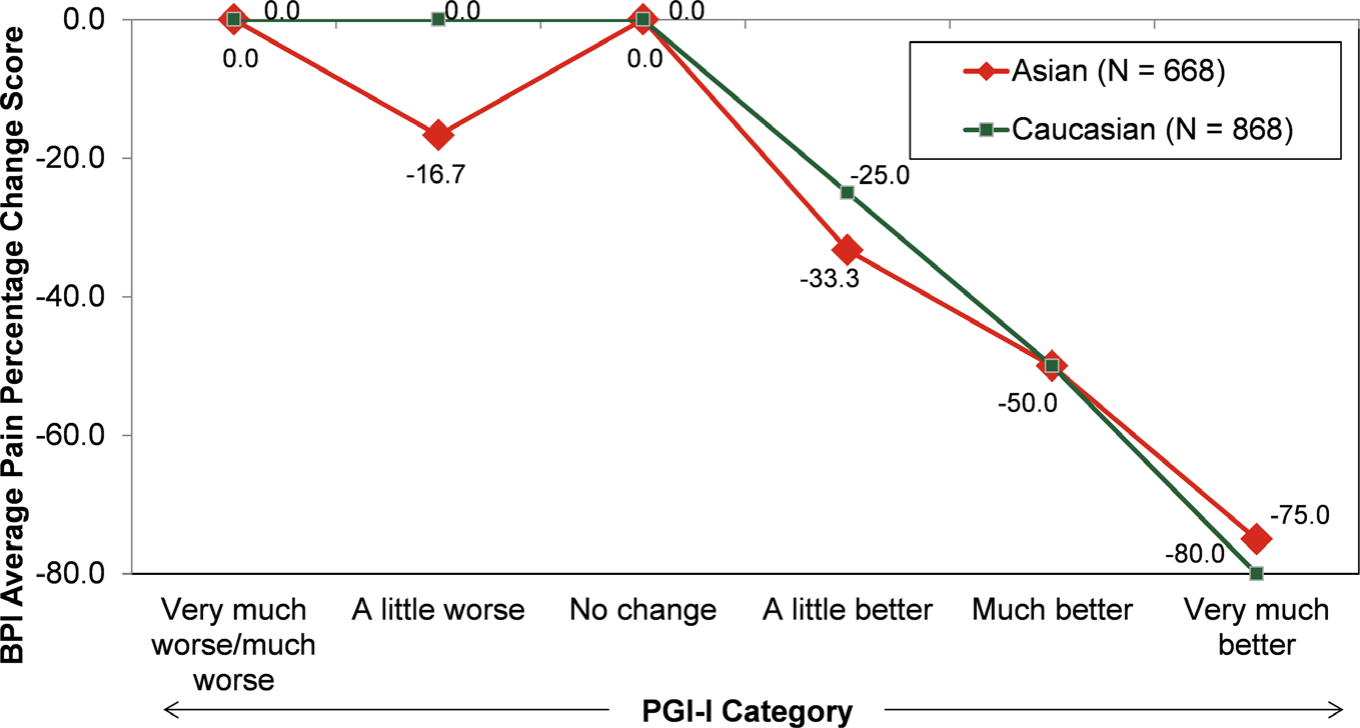
Reduction of Brief Pain Inventory (BPI) average pain scores from baseline to endpoint by Patient Global Impression of Improvement (PGI‐I) category according to race. N, number of subjects in analysis population. Data presented are median values.

**Figure 2:**
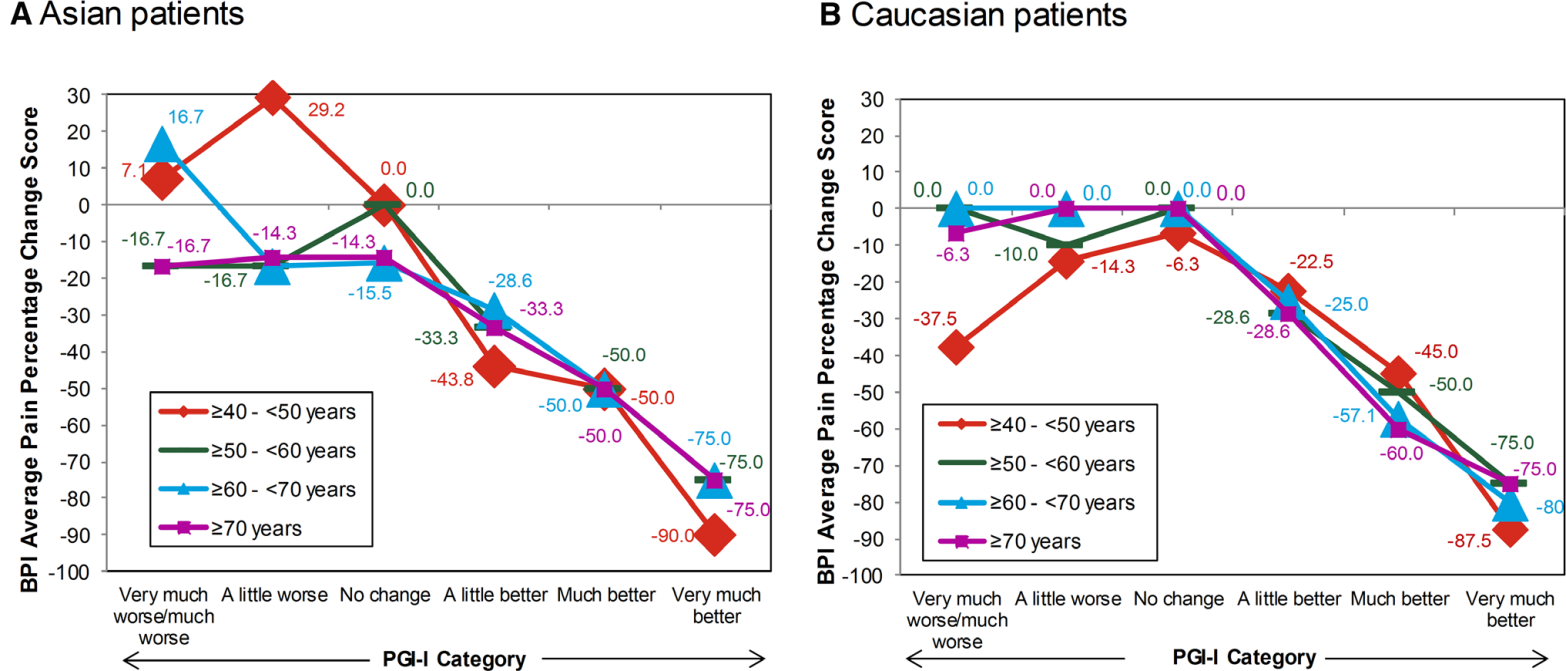
Reduction of Brief Pain Inventory (BPI) average pain scores from baseline to endpoint by Patient Global Impression of Improvement (PGI‐I) category according to age group in Asian (A) and Caucasian (B) patients. Data presented are median values.

**Figure 3:**
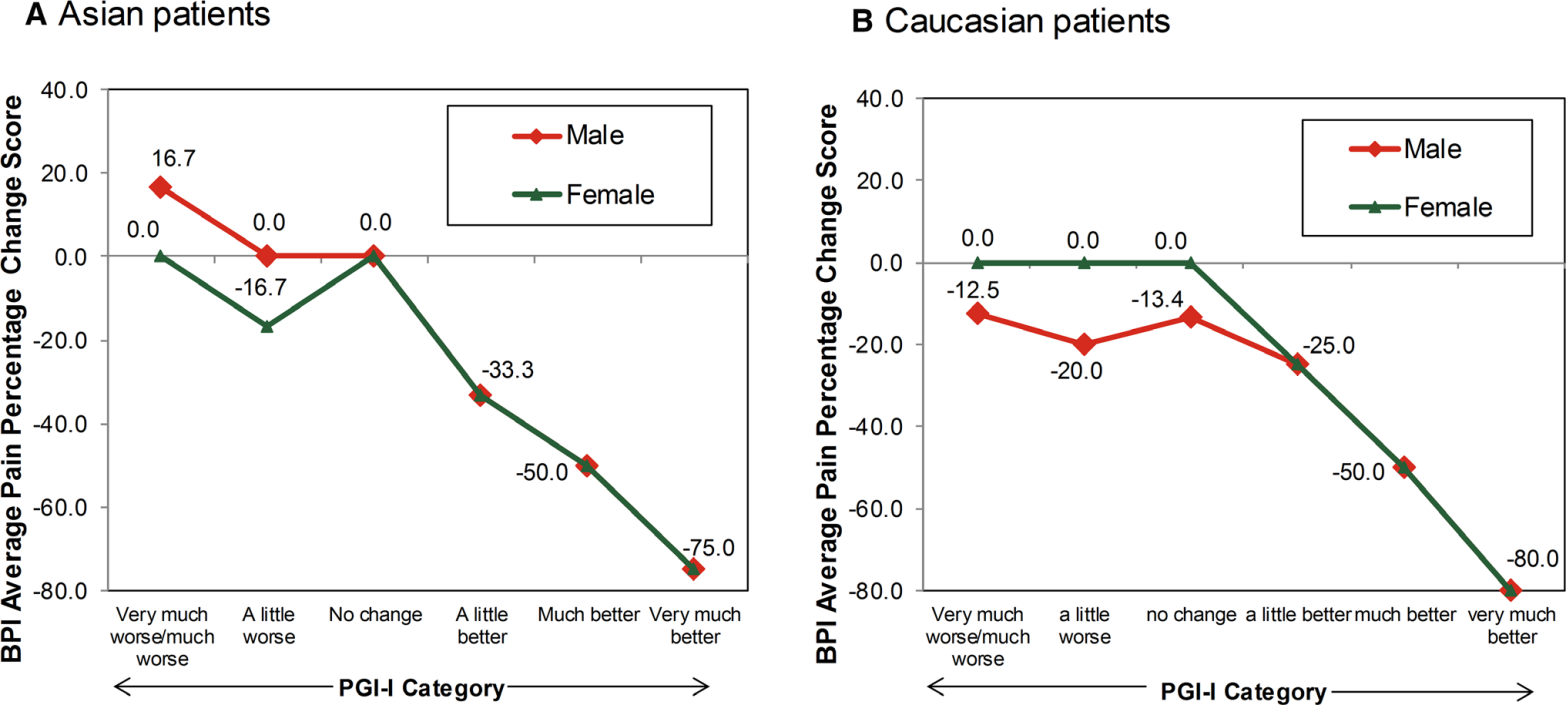
Reduction of Brief Pain Inventory (BPI) average pain scores from baseline to endpoint by Patient Global Impression of Improvement (PGI‐I) category according to gender in Asian (A) and Caucasian (B) patients. Data presented are median values.

**Figure 4:**
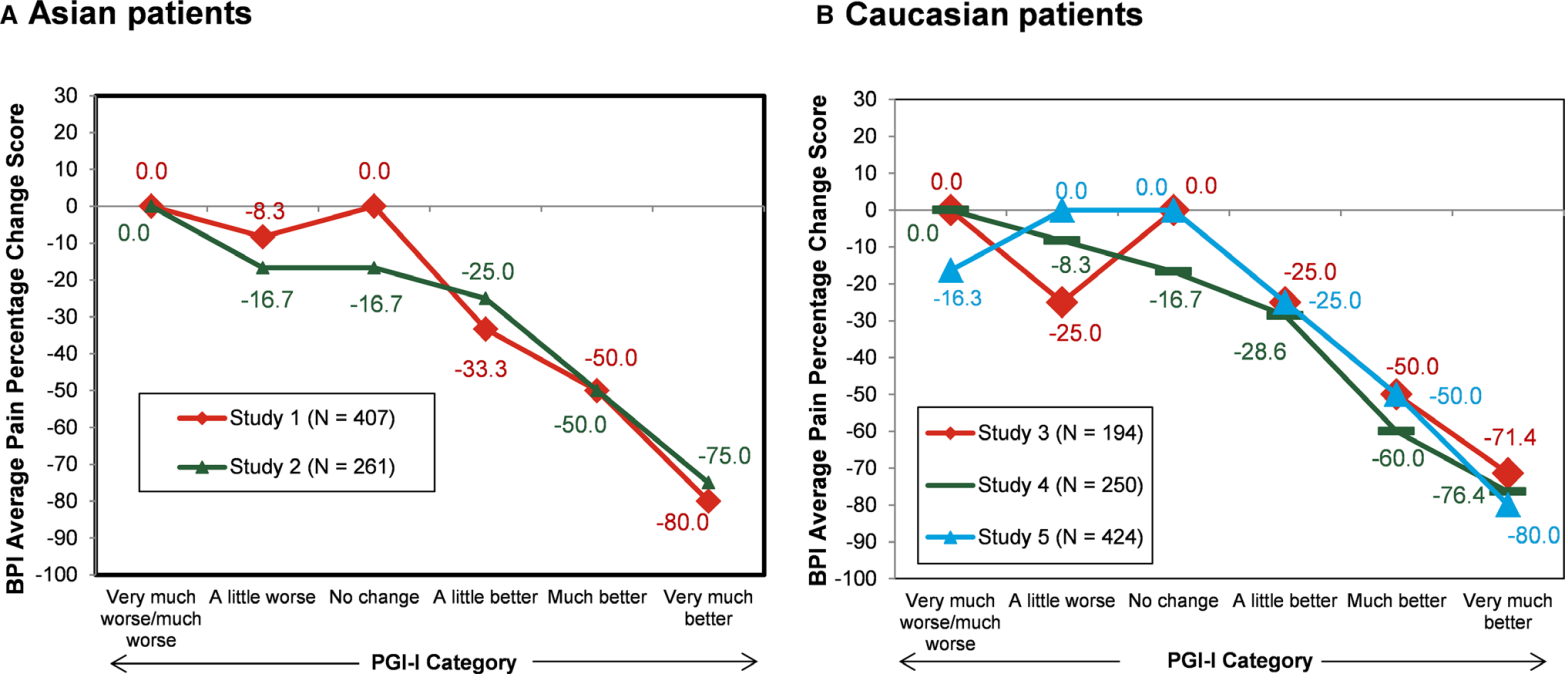
Reduction of Brief Pain Inventory (BPI) average pain scores from baseline to endpoint by Patient Global Impression of Improvement (PGI‐I) category according to study participation. N, number of subjects in analysis population for each study. Data presented are median values. Clinical trial identifiers: Study 1, NCT01931475; Study 2, NCT02248480; Study 3, NCT00408421; Study 4, NCT00433290; Study 5, NCT01018680.

**Figure 5:**
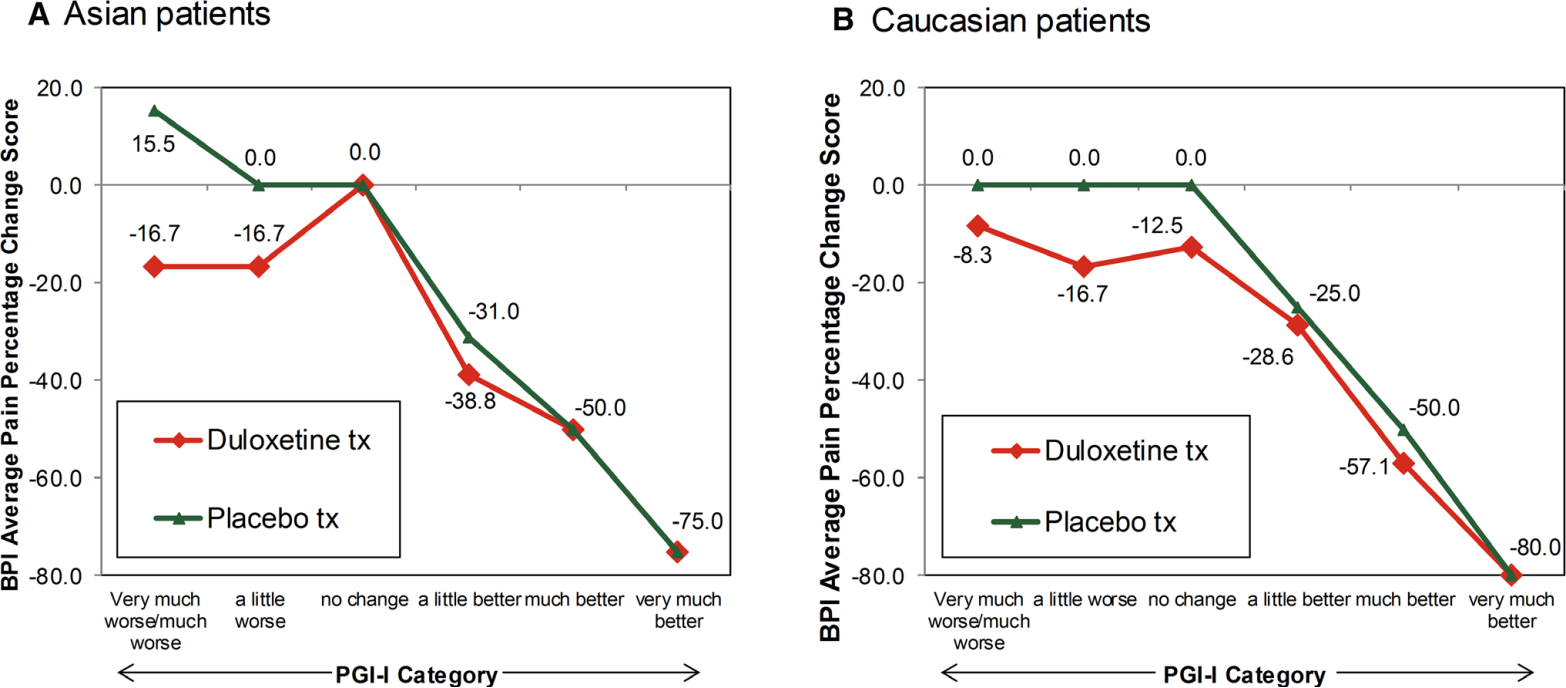
Reduction of Brief Pain Inventory (BPI) average pain from baseline to endpoint by Patient Global Impression of Improvement (PGI‐I) category according to treatment in Asian (A) and Caucasian (B) patients. tx, treatment. Data presented are median values.

**Figure 6:**
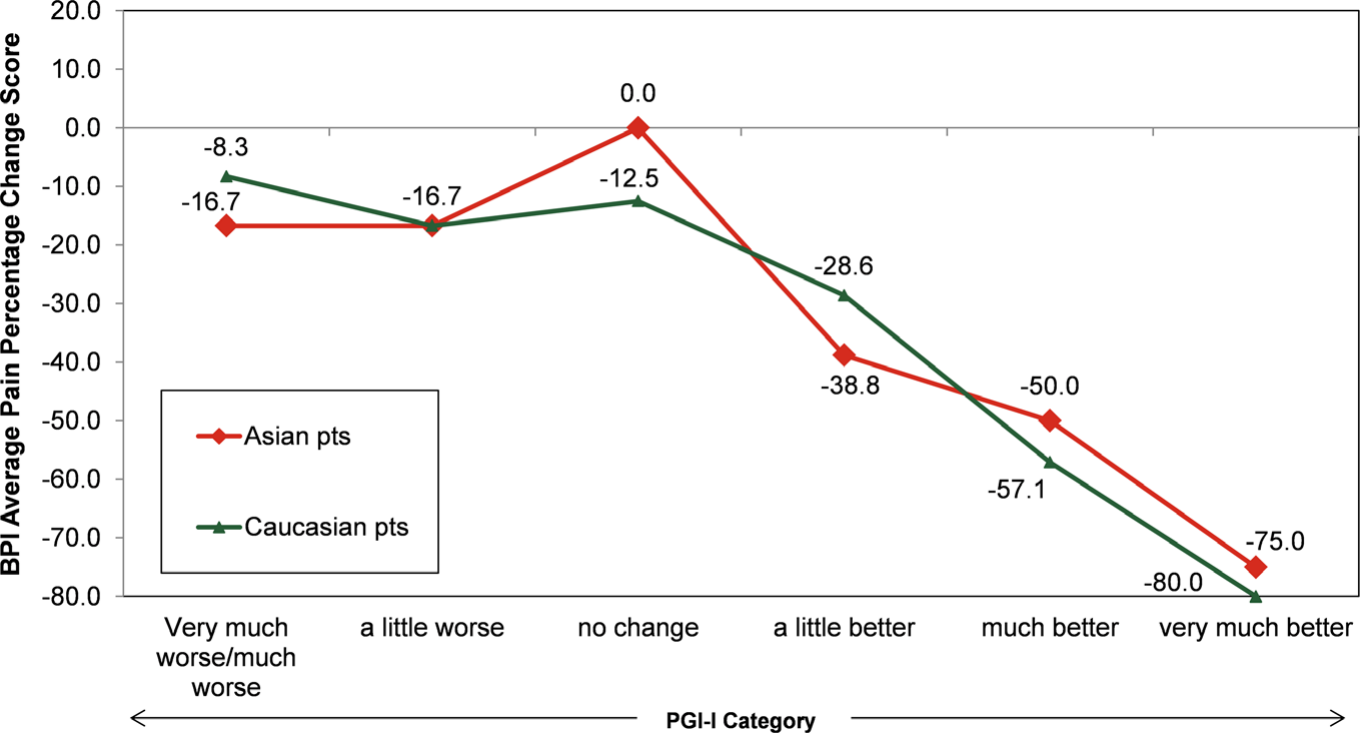
Reduction of Brief Pain Inventory (BPI) average pain from baseline to endpoint by Patient Global Impression of Improvement (PGI‐I) category according to race in duloxetine‐treated patients (*N* = 751, where Asian = 328; Caucasian = 423). Data presented are median values.

The ratings for percentage change from baseline for BPI average pain scores were similar across the 7 PGI‐I categories for Asian and Caucasian patients (Figure 1). Asian and Caucasian patients had nearly identical ratings for median percentage change associated with each level of the PGI‐I, indicating a consistent clinical interpretation of changes on the BPI average pain percentage change ratings, irrespective of ethnicity. The results for percentage change from baseline for BPI average pain scores were stratified by age (Figure 2), gender (Figure 3), study participation (Figure 4), and treatment (Figure 5) for Asian and Caucasian patients. For each stratifying factor, the patterns of median percentage change across PGI‐I categories are comparable between Asian and Caucasian patients. Overall, the relationship between BPI average pain percentage change and PGI‐I categories was nearly identical for Asian and Caucasian patients for each stratum. For each stratum, Asian and Caucasian patients had nearly identical median percentage change in pain ratings associated with each level of the PGI‐I, indicating a consistent clinical interpretation of changes on the BPI average pain percentage change in Asian and Caucasian patients, irrespective of age, gender, study participation, and treatment. Figure 6 shows the reduction of BPI average pain scores from baseline to endpoint by PGI‐I category according to race (Asian vs. Caucasian) in duloxetine‐treated patients. Overall, these results show that Asian and Caucasian patients had nearly identical median percentage change pain ratings associated with each level of the PGI‐I, indicating a consistent clinical interpretation of changes on the BPI average pain percentage change in duloxetine‐treated patients, irrespective of race.

Table 2 demonstrates specific values generated from the ROC analyses for both raw change and percentage change in the BPI average pain rating associated with 3 different levels of improvement as characterized by the PGI‐I (ie, “a little better” or higher; “much better” or higher; and “very much better”). The areas under the ROC curves for the raw BPI average pain change and percentage change are nearly identical for the definition of clinically important improvement in Asian and Caucasian patients. The sensitivity, specificity, and accuracy for the determination of the cut‐off values are also presented in Table 2. The ROC analysis results “much better” or “very much better” were −3 for the raw change in BPI average pain scores for both the Asian patients and Caucasian patients. The percentage change in BPI average pain was −40% for both Asian and Caucasian patients (see Table 2). These results show that CID was similar among Asian and Caucasian patients with OA.

**Table 2:** Receiver Operating Characteristic Analysis for Brief Pain Inventory (BPI) Average Pain by Race

BPI Average Pain	Clinically Important Change by PGI‐I	AUC	Change Cut‐off Point	Sensitivity (%)	Specificity (%)	Percentage Correct (%)
Asian (*N* = 650)						
Raw change	Very much better	0.829	−3.0	84.4	66.0	67.8
Raw change	Much or very much better	0.786	−3.0	64.7	78.1	72.8
Raw change	A little, much, or very much better	0.809	−2.0	72.3	77.0	73.4
Percentage change	Very much better	0.870	−63%	78.1	84.5	83.8
Percentage change	Much or very much better	0.806	−40%	80.6	66.8	72.3
Percentage change	A little, much, or very much better	0.815	−29%	71.7	78.3	73.2
Caucasian (*N* = 847)						
Raw change	Very much better	0.840	−3.0	89.5	66.6	69.7
Raw change	Much or very much better	0.843	−3.0	74.0	83.2	79.3
Raw change	A little, much, or very much better	0.827	−2.0	72.8	80.9	75.1
Percentage change	Very much better	0.884	−56%	84.2	78.7	79.5
Percentage change	Much or very much better	0.860	−40%	79.9	78.9	79.3
Percentage change	A little, much, or very much better	0.834	−30%	67.3	88.1	73.1

AUC, area under the curve; *N*, number of subjects in analysis population; PGI‐I, Patient Global Impression of Improvement.

## Discussion

The present analysis of pooled data from 5 studies of duloxetine in patients with chronic pain demonstrated a close association between changes in BPI average pain scores and PGI‐I scores in Asian and Caucasian patients. In Asian and Caucasian patients, the relationship is highly consistent across all analyzed studies, the treatments administered (duloxetine or placebo), and the patient demography characteristics (age and gender). The BPI average pain (percentage) reduction from baseline to endpoint was similar across 7 PGI‐I categories for Asian and Caucasian patients, regardless of age, gender, study, and treatment. The consistency of these results suggests that Asian and Caucasian patients interpret changes in BPI average pain scores and PGI‐I scores similarly, irrespective of age, gender, study, and treatment.

In the present analysis, close associations were found between BPI average pain ratings (raw and percentage change) and PGI‐I scores among Asian and Caucasian patients, as demonstrated by the area under the ROC. The raw levels of change in the BPI average pain scores associated with specific PGI‐I improvement levels could vary based on the baseline pain ratings. Therefore, the percentage change was calculated to adjust the baseline pain rating of each patient; hence, the relationship between the reported change in pain and the patients’ perception of improvement becomes more linear as the mean rating of the BPI average pain change and the PGI‐I categories becomes more consistent.

In Asian patients, a percentage change of 40% best correlated with our a priori definition of a CID, namely the PGI‐I category of “much better” or “very much better.” Similarly, a 63% reduction in pain from baseline correlated with the highest degree of improvement on the PGI‐I scale. In Caucasian patients, a percentage change of 40% best correlated with our a priori definition of a CID. Likewise, a 56% reduction in pain from baseline was associated with the highest degree of improvement on the PGI‐I. Also, raw change in BPI average pain scores for “much better” or “very much better” was −3 for the Asian patients and −3 for the Caucasian patients. These results indicate that the percentage and change cut‐off values for CID were similar in Asian and Caucasian patients with OA of the knee or hip. The present analysis results suggest that these values are suitable for use in the interpretation of clinical study results for the design and analysis of future clinical trials of chronic pain treatment. In comparing our results with those of other published studies^13^, ^21^, ^32^, ^33^, ^34^, ^35^, ^36^ in chronic pain comparing BPI average pain scores to a PGI‐I scale, our value of 40% in Asian and Caucasian patients for average pain is slightly higher but generally consistent. The published range in the level of change best associated with patient reporting of “much better” or “very much better” on a PGI‐I scale differs from 30% to 33%.^13^, ^21^, ^37^, ^38^, ^39^ This is also in line with the consensus statement released by IMMPACT in 2008.^14^ The slight difference between results in the present analysis and previous published studies is possibly due to variations in treatment durations and patient populations (particularly baseline pain severity scores).

The strength of the present analysis is the consistency observed across all 5 large duloxetine studies conducted in multiple countries with essentially identical measurement instruments and methodology. The common design among the studies eliminates many of the sources of variability, making the association between the change in the BPI average pain scores and PGI‐I scores less likely to have alternative explanations. The present analysis includes patients of different ethnicity (Asian and Caucasian patients). This may provide robust support for the external validity of these results. There are some limitations to the present analysis. The present analysis was performed only in a setting of OA; thus, these findings may not generalize to all chronic pain conditions. Additionally, the PGI‐I score may be affected by patients’ expectations of treatment, and patients’ responses to pain may be influenced by their perception of other improvements in their condition. The present analysis did not examine the effect of these other components, but the consistency of the demonstrated associations across patient populations, age, race, and sex improves the generalizability of our findings. Apart from the concept of symptomatic improvement in a patient's condition, the concept of satisfaction with care could also be used to assess a cut‐off point. However, satisfaction with care has a distinct theoretical concept and has not been included in most pain clinical trials, including those analyzed here.

The findings of the present analysis can help design future studies and estimate appropriate sample sizes of pain clinical trials. The presented data also facilitate the comparison of results across Asian and Caucasian patients, which helps to determine the value of a therapy in clinical practice. The findings of this study provide information about the range of BPI average pain scores associated with various degrees of global improvement (ie, PGI‐I category), irrespective of ethnicity, age, and gender, which can facilitate the evaluation of treatments for chronic pain.

## Conclusions

In the present analysis of 5 clinical studies of duloxetine in chronic pain due to OA, a consistent relationship between the change in BPI average pain scores and the PGI‐I category was demonstrated in Asian and Caucasian patients, regardless of age, gender, study, and treatment. Moreover, raw and percentage change cut‐off values for CID were similar in Asian and Caucasian patients with OA of the knee or hip. Overall, the present analysis concludes that the ratings for percentage change in BPI average pain by PGI‐I category and the cut‐off for CID were similar for the Asian and Caucasian populations. Applying these findings to future studies may help to standardize the definition of a CID in clinical trials of chronic pain therapies. Use of a standard outcome across chronic pain studies would greatly enhance the comparability, validity, and clinical applicability.

## Conflicts of Interest

All authors are Eli Lilly and Company employees, except for Li Yue. Hiroyuki Enomoto, Shinji Fujikoshi, Levent Alev, Yan Yolanda Cheng, and Vladimir Skljarevski are minor stakeholders. Li Yue and Jianing Wang have no conflicts of interest to declare.
